# Early childhood caries trends and surveillance shortcomings in the Czech Republic

**DOI:** 10.1186/1471-2458-12-547

**Published:** 2012-07-24

**Authors:** Erika Lenčová, Hynek Pikhart, Zdeněk Broukal

**Affiliations:** 1 Institute of Clinical and Experimental Dental Medicine - 1st Faculty of Medicine of the Charles University and General Teaching Hospital, Prague, the Czech Republic; 2 Department of Epidemiology and Public Health, University College London, London, UK

**Keywords:** Early childhood caries, Caries experience, Primary dentition, Oral health surveillance

## Abstract

**Background:**

Despite the decline in childhood caries prevalence, seen particularly in 1980s, in recent years there have been reports that the declining trend has stopped or even reversed in some countries. The aim of the study was to analyse data from previous epidemiological studies on early childhood caries in the Czech Republic, conduct a secondary analysis of trend in dental caries prevalence, and discuss issues related to national oral health surveillance.

**Methods:**

Since the 1990s, caries prevalence in preschool children was monitored by two independent bodies: Institute of Health Information and Statistics (IHIS) that conducted 5 cross-sectional surveys over the period 1994–2006, and Institute of Dental Research (IDR) that conducted 4 studies over the years 1998–2010. Both study series differed in methods of sample selection and approaches to examiner training. For the assessment of the caries prevalence trends, regression modelling was used for the following oral-health indicators: caries experience, mean number of teeth with untreated caries (dt) and percentage of caries-free children.

**Results:**

In both study series, a significant overall trend of declining caries experience and level of untreated caries, and an increasing trend of percentage of caries-free children was observed (p < 0.05). In IHIS studies, caries experience reduced from 3.5 to 2.7; dt reduced from 2.2 to 1.5 and a proportion of caries-free children increased from 23.9 to 42.2%. In IDR studies, caries experience reduced from 3.7 to 2.98; dt reduced from 2.5 to 2.1 and a proportion of caries-free children increased from 26.7 to 44.9%.

**Conclusions:**

Both study series identified a significant decline of caries prevalence particularly in the 1990s and early 2000s. By the end of the investigated period, flattening of the caries decline was observed. The positive trend was observed in the absence of any systematic preventive initiatives on a population level. With respect to the above the authors assume that in the Czech Republic there still is a potential for further caries reduction in preschool population. This, however, cannot be expected without any health policy interventions. Oral health surveillance in the Czech Republic should be promoted by competent regulatory authorities.

## Background

Dental caries is the most common chronic disease in childhood. If not managed properly, it may result in significant acute and chronic conditions, the most severe of which include bacteraemia and impaired development, not to mention high treatment costs and consequences to families and communities [1-3]. The American Academy of Pediatric Dentistry (AAPD) classifies early childhood caries (ECC) in a broad definition as ´the presence of one or more decayed (non-cavitated or cavitated lesions), missing (due to caries), or filled tooth surfaces in any primary tooth in a child 71-month-old or younger´[4]. It is complicated to compare global trends of early childhood caries prevalence mainly due to inconsistencies in the methodology of observational studies in individual countries. Despite the prevailing declining trend in dental caries, particularly seen in 1980s [5], some authors have reported that such a trend has stopped or even reversed for the primary dentition in recent years, particularly in countries that already had low caries prevalence in primary dentition [6-9]. It is well documented that the distribution of ECC in the population is skewed, with about one third of the examined population bearing most of the disease burden [10]. Cross-sectional epidemiological studies on ECC prevalence that employ standard epidemiologic measures, such as dmft index (d – number of teeth with untreated dental caries, m – number of teeth extracted due to dental caries, f – number of teeth with caries treated with a filling or crown) and a proportion of the population with intact dentition (dmft = 0) do not reflect the full scope of ECC´s impact on the society [3]. Nevertheless, they collect elementary data necessary for planning the most appropriate preventive interventions against ECC and for evaluating their effectiveness. Oral health data for monitoring disease patterns and trends over time represent an essential component of global oral health information systems established by WHO [11]. The aim of the study was to analyse primary data from previous epidemiological studies on the prevalence of early childhood caries in the Czech Republic, conduct a secondary analysis of trends in dental caries prevalence and discuss national oral health surveillance issues. The Czech Republic is also a country undergoing rapid social, economic and health-care transition and, as such, an interesting place to assess trends in dental caries in changing social settings.

## Methods

After political changes in the Czech Republic in 1989, data on caries experience of 5-year-olds were collected by two independent bodies: Institute of Health Information and Statistics (IHIS) and Institute of Dental Research (IDR). Data from the study series conducted by those two bodies were analyzed in the presented study.

### IHIS studies

In the years 1994, 1997, 2000, 2003 and 2006, five cross-sectional national surveys of caries experience in 5-year-olds were conducted by IHIS. The studies were supported by the Czech Ministry of Health as a part of national health monitoring programme. In each study, all general dental practitioners in the country were asked to collect caries experience data of all 5-year-old patients that would come for a dental appointment within one calendar month (April). To prevent doubling of the data in the case of repeated visits, each subject was included only once. With respect to the fact that providing the data was made mandatory by legislation, the response rate in each of the IHIS studies was almost 100%. No calibration exercise was performed and methodology was described in a brochure distributed to all examiners. Dental examinations were performed in dental offices under standard clinical conditions, x-ray examinations were not included. Dental caries detection threshold was a cavitated carious lesion as recommended by WHO [12]. Caries experience, as measured by the dmft index, was recorded in a standard WHO form distributed with the methodology leaflet. Completed forms were sent back to IHIS and processed centrally. Data analysis involved dmft descriptive statistics.

### IDR studies

IDR conducted national cross-sectional surveys on caries experience of 5-year olds in the years 1998, 2001, 2005 and 2010 as part of oral epidemiological research projects. In each study, a representative geographically stratified national sample of children in preschool nurseries aged more than 5 and less than 6 years was selected in compliance with the WHO manual for Oral health surveys [12]. The children were included in the study after their parents signed informed consent forms (ICF) agreeing to participation in the study. All studies were approved by the Institutional Ethics Committee. Dental examinations were conducted in nurseries using dental mirror, rounded probe and headlight. In each of the IDR studies, calibration exercise was carried out and a high inter-examiner reliability (Kappa values >0.80) was achieved. Dental caries was detected at the level of a cavitated lesion, and caries experience was recorded in the form of dmft index.

### Present study

For the assessment of caries prevalence trends observed by IHIS and IDR studies, regression modelling was used for each of the above described caries indicators, i.e. mean dmft per child, mean dt per child, and percentage of subjects with dmft = 0, separately in each group of studies (IDR and ECC). The significance threshold was set at p < 0.05.

## Results

Table 1 shows total number of study subjects, mean dmft value per child, mean number of teeth with untreated caries per child (dt value) and proportion of children with intact dentition (dmft = 0) for each wave of IHIS surveillance.

**Table 1:** IHIS studies: sample sizes and main results

	**1994**	**1997**	**2000**	**2003**	**2006**
Total number of study subjects	3383	3578	3186	3337	3561
Mean dmft per child	3.5	3.6	3.4	2.6	2.7
SE	0.06	0.06	0.06	0.06	0.06
Mean dt per child	2.2	2.2	1.8	1.5	1.5
SE	0.05	0.05	0.05	0.04	0.04
% of children with dmft =0	23.9	24.7	29.8	41.6	42.2

Table 2 shows total number of study subjects, overall response rate, mean dmft value per child, mean number of teeth with untreated caries per child (dt value) and percentage of subjects with dmft = 0 for each IDR study.

**Table 2:** IDR studies: sample sizes and main results

	**1998**	**2001**	**2005**	**2010**
Total number of study subjects	435	297	285	583
Overall response rate (%)	84.9	69.1	73.8	80.2
Mean dmft per child	3.7	3.3	2.8	2.9
SE	0.25	0.21	0.22	0.20
Mean dt per child	2.5	2.3	2.0	2.2
SE	0.19	0.18	0.19	0.15
% of children with dmft =0	26.7	31.30	51.2	44.9

**Figure 1:**
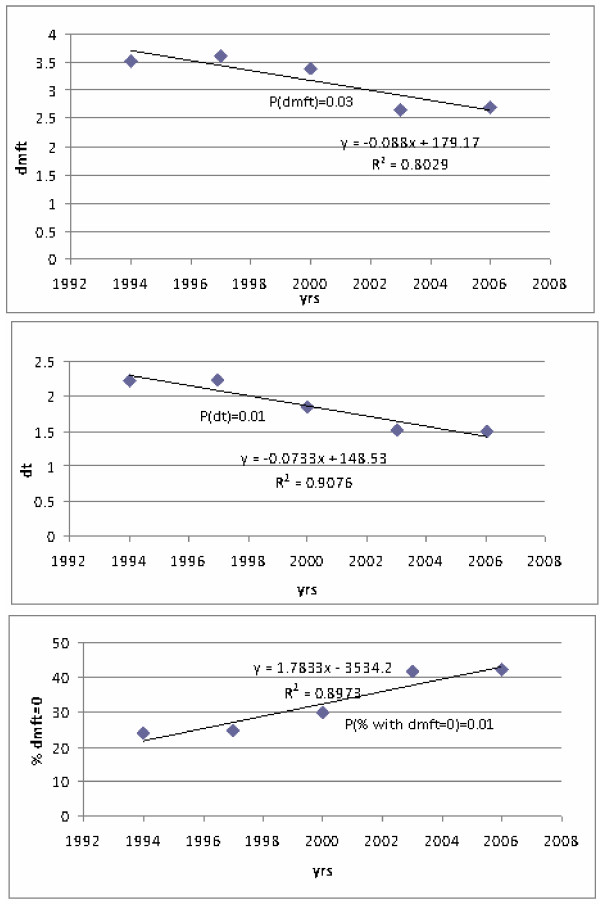
Regression trends of individual dental caries experience indicators, IHIS studies.

**Figure 2:**
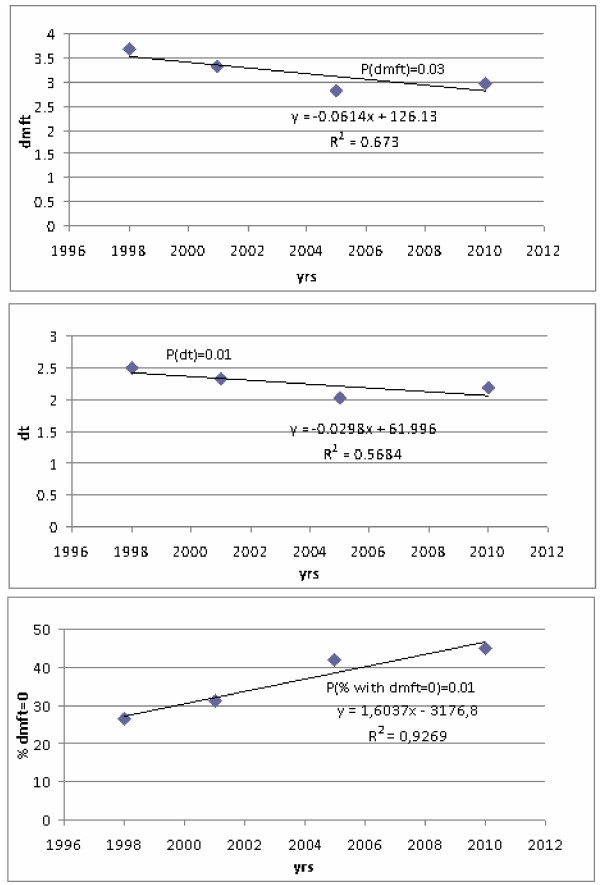
Regression trends of individual dental caries experience indicators, IDR studies.

The respective regression lines together with R2 and respective p-values are shown in Figures 1 and 2. In a series of IHIS studies conducted over years 1994–2006, a significant trend of declining mean dmft and mean dt value per child and increasing trend of percentage of caries-free children was observed (p < 0.05). Mean dmft value reduced from 3.5 to 2.7; mean dt values reduced from 2.2 to 1.5 and a proportion of caries-free children increased from 23.9 to 42.2%. In the IDR studies conducted over the years 1998–2010, a significant trend of declining mean dmft, mean dt value per child and increasing trend of percentage of caries-free children was observed (p < 0.05). Mean dmft value reduced from 3.7 to 2.98; mean dt values reduced from 2.5 to 2.1 and a proportion of caries-free children increased from 26.7 to 44.9%.

## Discussion

A positive trend of caries prevalence over a period 1994–2010 was observed both for national monitoring and smaller epidemiological studies. The two study series employed different methods of study sample selection (dental patients vs. subjects from a stratified national sample) and a different approach to examiner training (non-calibrated vs. calibrated examiners). Consistency observed in trends of individual parameters enhances the validity of the observed results. As it is evident from the data shown in Tables 1 and 2, the differences between the IHIS and IDR study results are generally small and they can be explained by methodological factors, e.g. by the differences between “patients” and “study subjects”. The study subjects recruited in the nurseries had higher proportion of intact teeth, but at the same time higher mean dmft and dt scores than patients examined in dental practices. This is probably related to the fact that the patients sought dental care predominantly not for preventive reasons, but because they were in need of dental treatment.

Detailed inspection of caries prevalence data shown in Tables 1 and 2 reveals that the decline in caries prevalence happened mainly in 1990s and early 2000s. By the end of the investigated period, both series of studies suggest possible flattening of the trend. This observation is consistent with reports showing stopping or reversal of this declining trend in childhood caries in some other countries [6-9]. Therefore, further surveys in the next few years would be needed to confirm whether this is a long-term trend or whether this was one-off event.

We can speculate on the reasons for the positive trend in caries experience. Over the recent years, there have been no systematic preventive initiatives against childhood caries implemented in the Czech Republic on a country- or regional level. Only isolated oral health programmes have been conducted in a few nurseries (such as oral hygiene training organized by dental students or programmes sponsored by dental companies). Dental prevention in preschool children has been conducted mostly only on individual level. Currently it has been a responsibility of parents to bring their child for dental check-up after the eruption of their first tooth; however, many of them neglect this responsibility. The underlying factors that might have influenced caries prevalence include children’s access to dental care and the availability of fluoride-containing products. These factors remained generally unchanged over the investigated period. As for the social and educational determinants influencing oral health, the standard of living (expressed by Gross Domestic Product per capita) and educational attainment of the Czech population (referring to the number of students in university education) were increasing steadily over this period, see Table 3[13,14].

**Table 3:** Selected social and educational determinants in the Czech Republic over the investigated period

	**1994**	**1997**	**2000**	**2003**	**2006**	**2009**
Gross Domestic Product per capita in USD	n/a	5 543	5 552	8 949	13 882	18 135
Number of students in university education	136 566	177 723	209 298	243 801	316 367	389 231

### Oral hearth surveillance issues

In 2006, due to a political decision, national oral health monitoring was terminated. The decision was probably guided by the cost-saving efforts of the government. In addition to that, at present, due to significant financial constraints on new research projects in the Czech Republic, no extensive regular oral health surveys can be planned. These factors significantly negatively influence national oral health surveillance.

The two presented study series both collected oral health data in a consistent way for more than a decade. Therefore they were chosen for the analysis of caries prevalence trend. Nevertheless, several potential sources of bias might have influenced their findings. IHIS studies recruited the subjects from patients who were actively seeking dental care. They involved non-calibrated examiners, but this was partially compensated by the fact that dental caries detection threshold was a cavitated lesion, and by large sample sizes. Nevertheless, limitations related to sample selection and non-calibrated examiners should not have influenced trends observed in the analysis, as the methods remained unchanged in all IHIS surveys; and thus the results from all 5 rounds were comparable with each other.

Inclusion of IDR study subjects based on parental informed consents interferes with random sample selection procedures. However, similarity of the results from both IHIS and IDR study series to some extent limits a possibility of differential bias related to response rates in IDR studies because response rates in IHIS were almost 100%.

In both IHIS and IDR studies, dental caries was detected using visual-tactile method at a cavity level as recommended by WHO. Choosing a cavitated carious lesion, i.e. a stricter criterion for the disease detection, reduces the incidence of false positive findings. However, it is currently generally accepted that such a detection threshold results in an increased number of false negative findings [15]. In case oral health surveillance is re-initiated in the Czech Republic, dental caries should be recorded at a pre-cavitation level as has been a common practice in epidemiological studies lately [16-19]. A geographically stratified national random sample used in IDR studies should be preferred to the sample recruited from dental patients.

In order to validly compare the ECC´s burden in different countries, it should be determined which definition better reflects the typical disease pattern, its severity and impacts. AAPD definition of ECC is significantly broad and sets no parameters for the disease severity. However, there is also a definition of severe ECC (S-ECC): ´in children younger than 3 years of age, any sign of smooth-surface caries is indicative of severe early childhood caries. From ages 3 through 5, 1 or more cavitated, missing (due to caries), or filled smooth surfaces in primary maxillary anterior teeth or a decayed, missing, or filled score of ≥4 (age 3), ≥5 (age 4), or ≥6 (age 5) surfaces constitutes S-ECC [20]´. This definition probably better reflects typical clinical picture of rampant caries. Another issue that should be addressed is a reference age group, representative for the population suffering from this disease and well accessible for surveying. The disease impact is particularly severe in children aged less than 3 years, therefore ECC data on this age group would be especially valuable. Nevertheless, in the Czech Republic, children attending nurseries (which generally admit children from the age of 3 to 6 years) are best accessible for the oral health surveys. Collecting caries prevalence data in dental patients aged less than 3 years by calibrated general dental professionals is another option. However, even though it is generally recommended that regular dental check-ups should be established with the eruption of the first primary tooth, only a small proportion of parents, most likely non-representative, bring their children to the dentist at that age. Therefore, creating a nationally representative sample of children aged less than 3 years seems to be an issue.

## Conclusions

The consistency observed in trends of individual parameters enhances the validity of the results in both sets of studies. Despite the observed decreasing trend of caries experience indicators in the examined cohorts, caries prevalence in Czech 5-year olds reported in this paper is still considerably higher than in other European countries [6,8]. It is also considerably higher than the targets set by WHO within the Health21 policy framework. Target 8.5 of this policy, related to the reducing of non-communicable diseases, stipulates that by the year 2020, at least 80% of children aged 6 years should be free of caries [21]. Therefore, the authors of this paper assume that in countries such as the Czech Republic, where the caries prevalence is still relatively high, there still seems to be a potential for caries reduction. This is documented by significant reduction in dental caries prevalence in the absence of any systematic preventive initiatives on a population level.

Further improvement of oral health of preschool population, however, cannot be expected without any health policy interventions. For the development and implementation of effective community preventive measures adequate to meet the needs of this population group, long-term monitoring of ECC trends is essential. National oral health data is required to assess oral health needs of the population, monitor oral health; plan effective intervention community programs and health policies; and evaluate the progress toward health objectives. Thus, systematic oral health surveillance in the Czech Republic should be promoted by competent regulatory authorities. This would help the policymakers obtain support for public oral preventive programs in an environment, which is highly competitive in terms of getting public resources [22], and to enable further long-term monitoring of childhood caries. Unfortunately, oral surveillance initiatives still remain a challenge in the Czech Republic.

## Abbreviations

AAPD, American academy of pediatric dentistry; ECC, Early childhood caries; dmft index, d – number of teeth with untreated dental caries, m – number of teeth extracted due to dental caries, f – number of teeth with caries treated with a filling or crown; dt, Number of teeth with untreated dental caries; WHO, World Health Organization; IHIS, Institute of health information and statistics; IDR, Institute of dental research; ICF, Informed consent form; S-ECC, Severe early childhood caries; GDP, Gross domestic product; USD, The United States dollar; n/a, Not available; SE, Standard error.

## Competing interests

The authors declare that they have no competing interests.

## Authors’ contributions

ZB and EL performed and/or collected data from the studies reported in the manuscript and processed the data. HP performed the statistical analysis. All authors contributed in writing the paper and approved the final draft.

## Pre-publication history

The pre-publication history for this paper can be accessed here:

http://www.biomedcentral.com/1471-2458/12/547/prepub
